# Becker's Nevus Syndrome in a Pediatric Female Patient

**DOI:** 10.1155/2016/3856518

**Published:** 2016-11-06

**Authors:** Sara Hernandez-Quiceno, Esteban Uribe-Bojanini, Juan Jose Ramírez-Jiménez, Maria Victoria Lopera-Cañaveral, Martin Toro-Ramos, Yuri Usuga-Arcila, Luis Correa-Londoño, Juan Camilo Martinez, Jennifer Monroy, Juan Manuel Alfaro

**Affiliations:** ^1^Pediatric Endocrinology Department, Universidad de Antioquia, Medellin, Colombia; ^2^Dermatology Department, Universidad CES, Medellin, Colombia; ^3^Dermatology Department, Universidad de Antioquia, Medellin, Colombia; ^4^Internal Medicine Department, Universidad de Antioquia, Medellin, Colombia; ^5^Laboratorio Clínico VID, Center for Dermatological Research (CIDERM), Universidad de Antioquia, Medellin, Colombia; ^6^Tissue Engineering and Cellular Therapy Group (GITTC), Universidad de Antioquia, Medellin, Colombia

## Abstract

Becker's nevus syndrome is part of the epidermal nevus syndromes and has been described with a phenotype that includes Becker's nevus, ipsilateral breast hypoplasia, and variable skeletal malformations. It is more frequent in males than in females (5 : 1) but is more relevant in females. The diagnosis is clinically based and the skin lesion must be present and no other numbered criteria have been established, but with more criteria being present the possibility of the diagnosis is higher. Regarding the treatment of breast hypoplasia, the use of antiandrogen medication has demonstrated adequate clinical response in a dose of 50 mg/day of spironolactone.

## 1. Introduction

Becker's nevus is an epidermal cutaneous hamartoma most commonly appearing in adolescents and more in men than women. Becker's nevus syndrome is a very rare syndrome with characteristics that include Becker nevus and hypoplasia of ipsilateral breast gland plus skeletal, muscular, or other skin disorders.

## 2. Case Report

**Figure 1:**
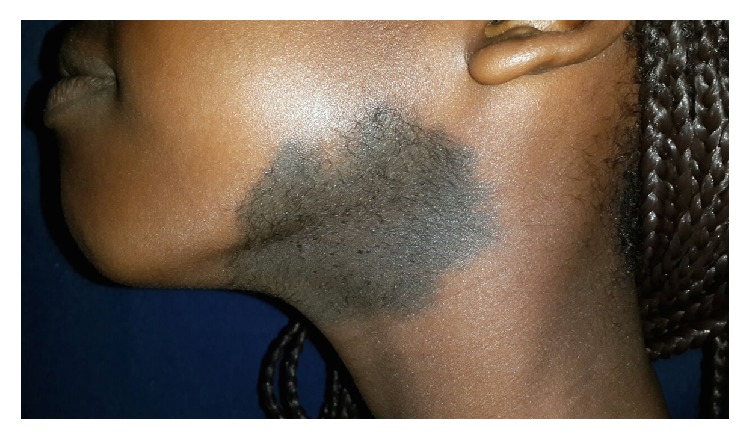
A pigmented congenital skin lesion of 3 × 4 centimeters with irregular borders and hypertrichosis in the left mandibular area.

**Figure 2:**
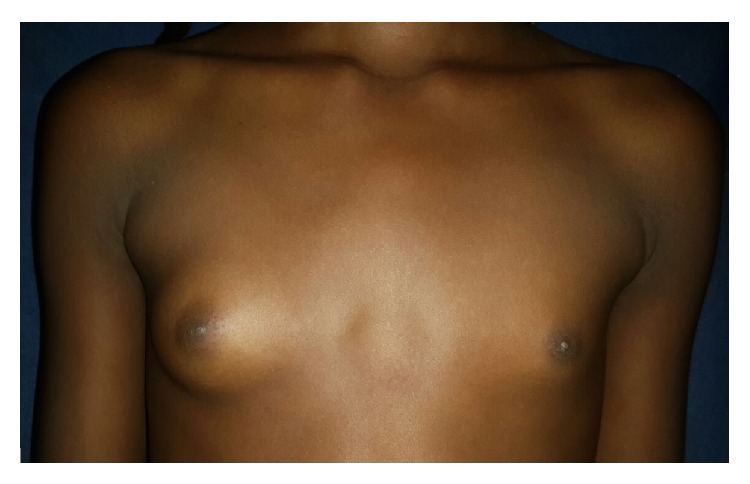
Pectus excavatum with right breast Tanner III-IV development and left breast Tanner II development plus marked hypoplasia.

**Figure 3:**
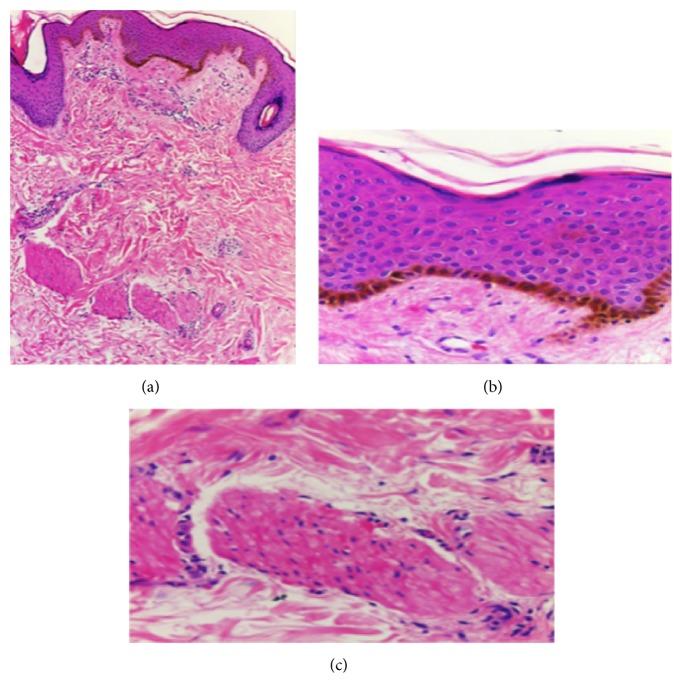
(a) Hyperkeratosis with irregular acanthosis and pigmentation of basal layer, with increase in the number of smooth muscle bundles (H & E ×10). (b) Pigmentation on the basal layer (H & E ×40). (c) Increase in the number of smooth muscle bundles (H & E ×40).

**Figure 4:**
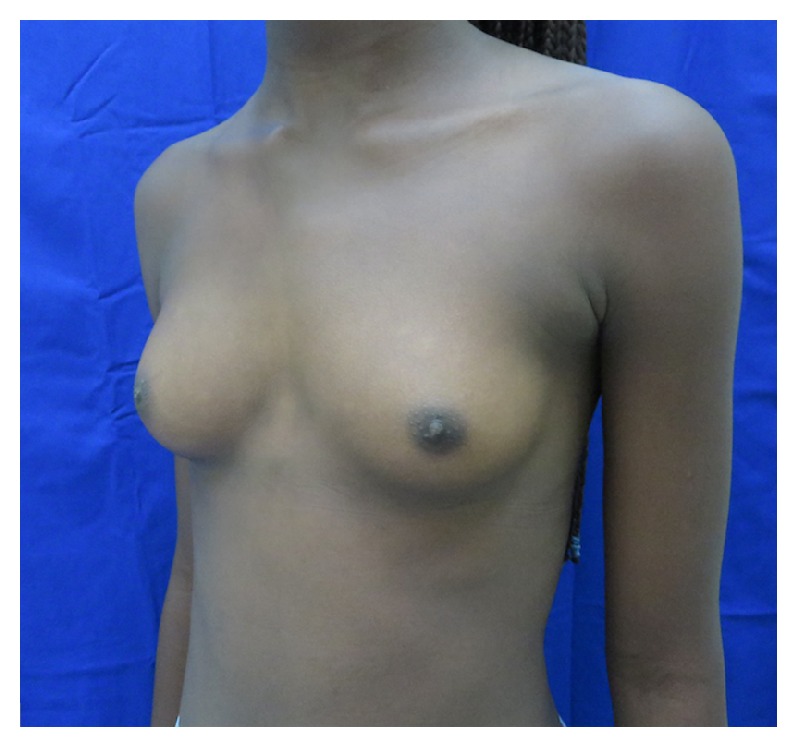
Picture showing improvement in left breast volume after therapy.

An 11-year-old female patient with prior medical history of umbilical hernia surgical correction consults because of left breast hypoplasia. Physical exam revealed a pigmented congenital skin lesion of 3 × 4 centimeters with irregular borders and hypertrichosis in the left mandibular area (Figure 1). In the thorax pectus excavatum was present, with right breast Tanner III-IV development and left breast Tanner II development plus marked hypoplasia (Figure 2). Biopsy of the skin lesion revealed an increase in the number of smooth muscle bundles and hair follicles and enlarged papillary crests with pigmentation in basal epidermis without signs of malignancy (Figure 3). Biopsy was compatible with Becker's nevus. Renal ultrasonography, renal function, and chest and spine X-ray were normal. Chest ultrasonography ruled out absence of mammary glands. Hormone levels (testosterone, prolactin, estradiol, FSH, LH, and TSH) were normal. Due to the combination of Becker's nevus, unilateral breast hypoplasia, umbilical hernia, and pectus excavatum, the diagnosis of Becker's nevus syndrome was established. The patient responded to spironolactone therapy with an outstanding improvement in left breast development (Figure 4).

## 3. Discussion

Becker's nevus (BN) is an epidermal cutaneous hamartoma first described in 1949 by Becker [1]. The common clinical presentation is multiple macules associated with hypertrichosis and hyperpigmentation in trunk or shoulder with a unilateral distribution. It can also present as nonhairy BN. Most studies in adults show a five times greater incidence in men than women, but few other studies have revealed a female predominance [2]. The higher incidence in men reported in adult case studies has not been found in children [3]. Histological evaluation reveals acanthosis, epidermic hyperpigmentation, papillomatosis, arrector pili muscle hyperplasiam and melanophages in dermis [4]. Despite the hyperpigmentation, BN is not included in the melanocytic lesions and it is a considered a particular form of epidermal nevi.

In the past, various authors had associated BN with other musculoskeletal disorders, but in 1997 Happle and Koopman described Becker's nevus syndrome (BNS) [5]. BNS is a very rare syndrome with characteristics that include Becker nevus and hypoplasia of ipsilateral breast gland plus skeletal, muscular, or other skin disorders. It is included in the group of epidermal nevus syndromes, with Proteus syndrome, nevus comedonicus syndrome, phakomatosis pigmentokeratotica, nevus sebaceous syndrome, and CHILD syndrome [5, 6]. The diagnosis is clinical and must include the presence of Becker's nevus and other skin, muscle, and/or skeletal disorders are also necessary, which may include ipsilateral hypoplasia of shoulder or arm, ipsilateral breast hypoplasia, supernumerary nipple, facial asymmetry, skin hypoplasia of temporal region, spina bifida, spinal fusion, pectus carinatum or excavatum, scoliosis, lipodystrophy, stress fractures, accessory scrotum, contralateral hypoplasia of labia minora, and umbilical hernia [7, 8]. The pathogenesis remains unclear; most cases occur sporadically, but familiar involvement may happen due to incomplete penetrance of autosomal dominant inheritance. Androgens have been involved due to the greater presentation in adolescence and men plus the association with hypertrichosis, acne, and scrotum abnormalities [9]. Since androgens are also related to bone, muscle, and hair development, this might play a role in the pathogenesis of the other systemic alterations. BN lesions have greater number of androgen receptors compered to normal skin; this suggests that BNS should be considered part of androgen receptor hypersensitivity syndrome spectrum. Breast hypoplasia may be explained by the counterbalance effect of androgens that decrease the estrogens effect [10].

No specific treatment for BNS has been described; since BN lesions are usually large, surgical treatment is limited. Treatment with Q-switched ruby (694 nm) and Erbium: YAG lasers have had satisfactory results. Also various successful depilation techniques can be used for cases with hypertrichosis [11]. A satisfactory response with a significant reduction of hyperpigmentation was reported with topical flutamide, a nonsteroidal antiandrogen [12]. Spironolactone is an antiandrogen used in other dermatological diseases due to the hormonal action. In relation to breast hypoplasia, its action is not fully understood but has been proposed to respond to negative feedback of androgen receptors. In dosage of 50 mg/day, improvement of breast hypoplasia has been described [13].

## 4. Conclusion

The case presented of an 11-year-old female patient, with a dermatological lesion clinically and histologically consistent with BN associated with previous umbilical hernia, ipsilateral breast hypoplasia, and pectus excavatum, meets the criteria described for Becker's nevus syndrome. The patient had an excellent response to spironolactone therapy regarding breast volume; this is consistent with other cases reported previously. Literature also supports that patients with BN should be evaluated for associated abnormalities of BNS.
